# Tracking of accelerometry-measured physical activity during childhood: ICAD pooled analysis

**DOI:** 10.1186/1479-5868-9-68

**Published:** 2012-06-07

**Authors:** Soyang Kwon, Kathleen F Janz

**Affiliations:** 1 Consortium to Lower Obesity in Chicago Children, Children’s Memorial Hospital, 2300 Children's Plaza, Box #157, Chicago, IL, 60614, USA; 2 Departments of Health and Human Physiology, University of Iowa, E130 Field House, Iowa City, IA, 52242, USA; 3 Epidemiology, University of Iowa, E130 Field House, Iowa City, IA, 52242, USA

**Keywords:** Stability, Objective measure, Exercise, Adolescents, Longitudinal

## Abstract

**Background:**

Understanding of physical activity (PA) tracking during childhood is important to predict PA behaviors and design appropriate interventions. We compared tracking of PA according to PA level and type of day (weekday/weekend) in a pool of five children’s cohort studies.

**Methods:**

Data from ALSPAC, CLAN, Iowa Bone Development Study, HEAPS, PEACH were extracted from the International Children’s Accelerometry Database (ICAD), resulting in 5,016 participants with age, gender, and accelerometry data at both baseline and follow-up (mean age: 10.3 years at baseline, 12.5 years at follow-up). Daily minutes spent in moderate- and vigorous-intensity PA (MVPA) and vigorous-intensity PA (VPA) was categorized into quintiles. Multinomial logistic regression models were fit to predict follow-up (M)VPA from baseline (M)VPA (reference: 20- < 80%tile), age at follow-up, and follow-up duration.

**Results:**

For the weekday, VPA tracking for boys with high baseline VPA was higher than boys with low baseline VPA (ORs: 3.9 [95% CI: 3.1, 5.0] vs. 2.1 [95% CI: 1.6, 2.6]). Among girls, high VPA was less stable when compared low VPA (ORs: 1.8 [95% CI: 1.4, 2.2] vs. 2.6 [95% CI: 2.1, 3.2]). The pattern was similar for MVPA among girls (ORs: 1.6 [95% CI: 1.2, 2.0] vs. 2.8 [95% CI: 2.3, 3.6]). Overall, tracking was lower for the weekend.

**Conclusions:**

PA tracking was higher on the weekday than the weekend, and among inactive girls than active girls. The PA “routine” of weekdays should be used to help children establish healthy PA patterns. Supports for PA increase and maintenance of girls are needed.

## Background

Although the absolute level of physical activity (PA) decreases during childhood and adolescence [1], PA behaviors are presumed to be habitual. That is, children have a tendency to maintain their rank of PA within a group over time. This phenomenon is known as tracking [2,3]. Tracking of low levels of PA has deleterious health implications, e.g., the chronic effects of low PA contribute to an increased risk of obesity and poor cardiometabolic profiles [4,5]. Whereas, tracking of high levels of PA is associated with metabolic health benefits presumably due to the accumulated effects of PA. Understanding of PA tracking during childhood is important to predict PA behaviors and design PA interventions. From a public health perspective, PA interventions should be designed to “untrack” low PA [6] and support tracking of high PA. Evidence on that a PA lifestyle is sustainable from childhood to adolescence supports PA promotion interventions for young children and comprehensive K-12 school physical education aimed at an active lifestyle.

A review by Telama [7] on tracking of PA suggested low to moderate tracking during childhood and adolescence. However, most of the reviewed studies used subjective measures or a small sample size, both of which limited the investigators’ ability to examine specific characteristics of PA. PA patterns are likely to differ based on their social context (e.g., school vs. home) or type of day (weekday vs. weekend) [8]. Yet, these attributes are rarely considered in the tracking studies. Furthermore, most tracking studies have examined overall tracking, and few have focused on children with low-levels of PA who are at a great risk of poor metabolic health. In this current study, we conducted a pooled analysis using accelerometry-measured PA data from five cohort studies to determine whether the magnitude of PA tracking differs between 1) weekday and weekend and 2) low-active and high-active children.

## Methods

### Study sample

Secondary data analysis was conducted using the International Children’s Accelerometry Database (ICAD). The ICAD is an archive of anonymized ActiGraph accelerometry data, predictors of PA, and/or associated phenotypic and socio-demographic data from 20 studies of 3 to 18 year-old children worldwide. Detailed information on the design and protocols of the ICAD project can be found elsewhere [9]. For the current analysis, we extracted data of seven cohort studies which had collected accelerometry data at ≥ 2 time-points (waves) from the ICAD: Avon Longitudinal Study of Parents and Children (ALSPAC), UK; Children Living in Active Neighourhoods (CLAN), Australia; Danish European Youth Heart Study (EYHS), Denmark; Healthy Eating and Play Study (HEAPS), Australia; Iowa Bone Development Study (IBDS), US; Personal and Environmental Associations with Children’s Health (PEACH), UK; and Portuguese EYHS, Portugal. The CLAN had three waves (median age: 10.9 year at wave 1, 13.9 years at wave 2, and 15.6 years at wave 3) and the IBDS had four waves (median age: 5.6 years at wave 1, 8.6 year at wave 2, 11.0 years at wave 3, and 13.0 years at wave 4). To align the median age and the age range with other study samples, data from waves 1 and 2 for the CLAN and from waves 3 and 4 for the IBDS were included in this report. While the Danish and Portugal EYHS had a six-year follow-up period, the other five studies had a follow-up period of 1–3 years; therefore, we excluded EYHS data and focused on short-term tracking. Study designs and data collection procedures have previously been described for individual studies: ALSPAC [10], CLAN [11], IBDS [12], HEAPS [13], and PEACH [14]. Because the ICAD dataset is anonymous data source, Human Subject Committee did not review this pooled analysis.

### Accelerometry data reduction

The data reduction process from raw ActiGraph data was performed by the ICAD group [9]. All raw data were analyzed using KineSoft version 3.3.20 (KineSoft, Saskatchewan, Canada; http://www.kinesoft.org). All accelerometry files from participating study groups were reintegrated to 60 second epochs. If a period of 60 minutes of consecutive zeros, allowing for 2 minutes of non-zero interruptions, was encountered anywhere in the data array, accelerometers were considered not worn and the data were not analyzed.

The inclusion criteria for accelerometry data for this report were a valid wear time of ≥ 10 hours per day and ≥ 3 days at each of baseline and follow-up. Moderate intensity was defined as 2,296 to 4,011 accelerometer counts per minute [15,16]. Vigorous intensity was defined as ≥ 4,012 accelerometer counts per minute [15,16]. Daily minutes spent in moderate- to vigorous intensity PA (MVPA—minutes/day) and vigorous-intensity PA (VPA—minutes/day) were the PAmeasures.

### Statistical analysis

All analyses were performed using the SAS 9.2 version (Cary, NC). Data analysis included participants with age, gender, and valid accelerometry data at both baseline and follow-up. If MVPA accelerometry data were > 6 hours/day, the individual was considered as an outlier and excluded in the data analysis.

#### Study-specific analysis

**Figure 1:**
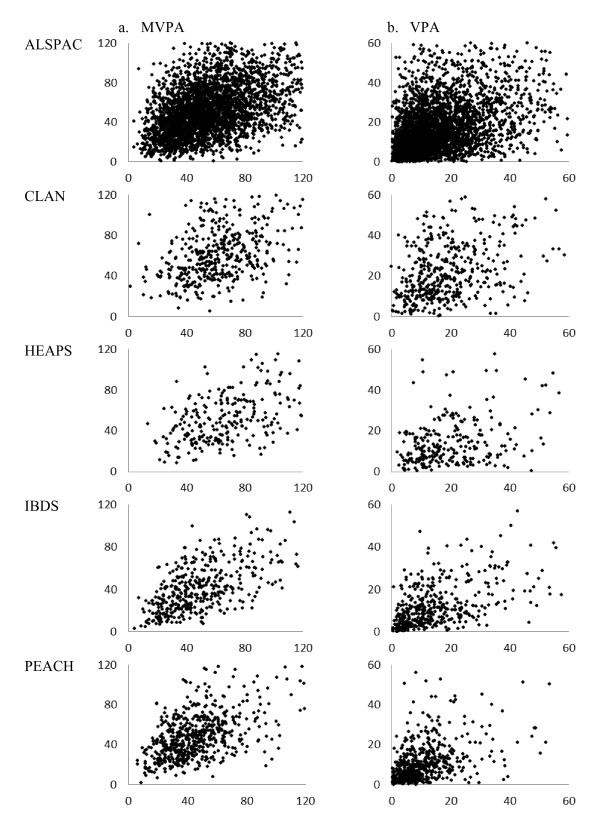
**Scatter plots of moderate- and vigorous-intensity physical activity by cohort studies. ***Note. * X axis = baseline (minutes/day), Y axis = follow-up (minutes/day). MVPA, time spent in moderate- to vigorous-intensity physical activity; VPA, time spent in vigorous-intensity physical activity.

To determine if a pooled analysis was appropriate, descriptive analyses of PA and tracking levels were performed for individual studies. Scatter plots of follow-up PA outcome over baseline PA outcome were examined. Although median MVPA and VPA varied across the studies (Table 1), the relationship between baseline and follow-up PA outcomes were similar (Figure 1). The overall homogeneity in the relationship indicated that a pooled analysis was appropriate. However, because the distribution of PA outcomes somewhat varied across the studies and waves, we used study- and wave-specific cut-points to categorize PA outcomes in pooled analysis.

**Table 1:** Characteristics of participants by cohort studies

	**Avon Longitudinal Study of Parents and Children (ALSPAC)**	**Children Living in Active Neighourhoods (CLAN)**	**Healthy Eating and Play Study (HEAPS)**	**Iowa Bone Development Study (IBDS)**	**Personal and Environmental Associations with Children’s Health (PEACH)**
**Country**	United Kingdom	Australia	Australia	United States	United Kingdom
**Source population**	Birth cohort recruited from a Bristol health district	Students in Melbourne public schools	Students in Melbourne public and catholic schools	Birth cohort recruited from Iowa hospitals	Students in Bristol public schools
**Accelerometry data files,**^**a**^**follow-up/baseline, n (%)**	4102/6085 (67%)	511/1162 (44%)	327/1390 (24%)	456/528 (86%)	889/1269 (70%)
**Sample,**^**b**^**n**	3324	444	259	415	579
**Compliance rate,**^**c**^**%**	81	87	79	91	65
**Gender**					
Boys, %	46.8	47.5	52.1	50.1	41.6
Girls, %	53.2	52.5	47.9	49.9	58.4
					
Baseline	All	Jul-Dec	Feb-Nov	Sep-Nov	Sep-Jul
Follow-up	All	Jul-Nov	May-Nov	Sep-Nov	Oct-Jul
					
**Follow-up duration, years**	2.1 (2.0, 2.3)	3.0 (2.9, 3.0)	3.0 (3.0, 3.1)	2.0 (2.0, 2.0)	1.1 (1.0, 1.1)
**Ages, years**					
Baseline	11.7 (11.6, 11.8)	10.9 (6.3, 11.6)	6.4 (5.8, 10.9)	11.0 (10.9, 11.2)	10.9 (10.6, 11.2)
Follow-up	13.8 (13.8, 13.9)	13.9 (9.2, 14.6)	9.6 (9.0, 13.9)	13.0 (12.9, 13.2)	12.0 (11.7, 12.3)
**Height, m**					
Baseline	1.50 (1.46, 1.55)	1.42 (1.20, 1.51)	1.24 (1.16, 1.44)	1.49 (1.43, 1.54)	1.45 (1.40, 1.50)
Follow-up	1.63 (1.58, 1.68)	1.60 (1.39, 1.68)	1.44 (1.34, 1.62)	1.62 (1.56, 1.66)	1.51 (1.46, 1.57)
**Weight, kg**					
Baseline	41.4 (36.2, 48.6)	35.5 (23.7, 43.8)	27.0 (21.7, 37.7)	41.8 (35.2, 51.0)	37.0 (33.0, 44.0)
Follow-up	52.6 (46.8, 60.2)	51.1 (35.3, 61.1)	41.0 (31.7, 53.6)	53.7 (45.4, 64.1)	42.8 (37.6, 50.8)
					
Baseline	7 (6, 7)	6 (5, 6)	5 (4, 6)	5 (4, 5)	5 (4, 6)
Follow-up	6 (5, 7)	6 (5, 6)	5 (4, 6)	5 (4, 5)	5 (4, 6)
					
Baseline	13.3 (12.8, 13.8)	13.1 (12.4, 13.9)	12.7 (12.0, 13.5)	12.8 (12.5, 13.5)	13.0 (12.4, 13.6)
Follow-up	13.5 (12.8, 14.1)	13.6 (12.9, 14.2)	13.2 (12.4, 13.9)	13.1 (12.6, 13.8)	13.3 (12.6, 14.0)
					
Baseline	52.3 (37.1, 70.9)	61.2 (47.7, 78.2)	67.4 (48.8, 88.7)	45.6 (32.6, 63.8)	43.3 (30.9, 57.7)
Follow-up	48.5 (33.3, 68.8)	59.1 (43.1, 79.0)	49.0 (33.8, 68.7)	40.0 (25.2, 57.3)	45.5 (32.2, 62.1)
					
Baseline	13.0 (7.6, 22.1)	17.2 (11.0, 26.0)	17.6 (10.8, 28.0)	12.4 (6.4, 22.6)	8.2 (4.5, 14.0)
Follow-up	14.0 (7.2, 24.4)	19.3 (11.4, 33.0)	11.3 (6.0, 19.2)	10.2 (5.0, 18.3)	8.0 (3.8, 14.2)

MVPA, moderate- to vigorous-intensity physical activity; VPA, vigorous-intensity physical activity.

^a^The number of accelerometry data files at follow-up was obtained from the number of participants who had accelerometry data files at follow-up among those who had accelerometry data files at baseline in the ICAD dataset.

^b^Sample size was the number of participants who had age, gender, and valid accelerometry data (≥ 10 hours/day and ≥ 3 wear days) at both baseline and follow-up.

^c^Compliance rate was calculated as sample size divided by the number of participants who had accelerometry data files at both baseline and follow-up.

#### Pooled analysis

Each study was weighted based on its sample size, so that each study contributed equally to the pooled analysis. The descriptive analyses for accelerometer outcomes were conducted stratified by gender, wave, and the type of day (weekday/weekend). Categorical variables of MVPA and VPA were created based on study-, wave-, and gender-specific quintile cut-points of daily minutes of MVPA and VPA. For type of day-specific analysis, quintile cut-points for each of weekday and weekend were used. This approach allowed an individual participant to be assigned into different rank categories of MVPA and VPA for weekday/weekend combined, weekday, and weekend.

Weighted kappa coefficients were estimated for measuring agreement of baseline and follow-up quintile categories for each of MVPA and VPA [17]. Due to the absence of a criterion-based cut-point for low accelerometry-determined PA, the lowest quintile of MVPA was considered low MVPA (inactive). The highest quintile of MVPA was regarded as high MVPA (active). The lowest quintile of VPA was considered as low VPA, and the highest quintile as high VPA.

Gender-specific multinomial logistic regression models were fit to examine stability of MVPA. Predictors included centered age at follow-up (years; continuous), follow-up duration (years; continuous), MVPA at baseline (<20%tile, 20 to <80%tile, and ≥80%tile; reference group: 20 to <80%tile). The dependent variable was MVPA at follow-up (<20%tile, 20 to <80%tile, and ≥80%tile; reference group: 20 to <80%tile). Shift to high or low PA of the 20 to <80%tile group was considered as neutral and served as the reference. Odds ratios (ORs) and 95% confidence intervals (CIs) were obtained from the models. The modeling was repeated for VPA as well as stratified by type of day. Biological maturity was not considered because accounting for differences in the timing of biological maturity has been reported to have little effect on tracking PA [18].

## Results

Excluding 50 individuals as outliers (41 individuals for ALSPAC, 7 individuals for CLAN, and 2 individuals for HEAPS), the final data analysis included 5,021 participants. Table 1 presents characteristics of participants of the five cohort studies. The original participation rates and loss to follow-up for each study were not available. However, based on accelerometry data files within the ICAD dataset, attrition and compliance rates (defined as the number of participants who had valid accelerometry data divided by the number of those who had accelerometry data files at both baseline and follow-up) are presented in Table 1. The race information for the CLAN and HEAPS was not available. Most participants in the other studies were Caucasian---96% of the participants in the ALSPAC, 95% in the IBDS, and 88% in the PEACH.

In a pooled sample, the mean age was 10.3 years (95% CI: 10.2, 10.3) at baseline and 12.5 years (95% CI: 12.5, 12.6) at follow-up. The mean follow-up duration was 2.1 years (95% CI: 2.1, 2.1). On average, girls engaged in approximately 20 minutes/day less MVPA than boys (*P* < 0.01; Table 2). Compared weekdays, MVPA and VPA were lower on weekends in boys and girls (*Ps* < 0.05).

**Table 2:** Means of physical activity levels. Pooled analysis

	**MVPA**		**VPA**	
	**Boys (n = 2349)**	**Girls (n = 2672)**	**Boys (n = 2349)**	**Girls (n = 2672)**
		Mean (95% confidence interval)		
**Weekday/weekend combined, minutes/day**				
Baseline (n = 5021)	69.1 (68.0, 70.3)	47.4 (46.6, 48.2)	21.0 (20.4, 21.7)	13.0 (12.6, 13.4)
Follow-up (n = 5021)	62.5 (61.3, 63.6)	43.8 (43.0, 44.7)	20.1 (19.4, 20.8)	12.6 (12.1, 13.1)
**Weekday, minutes/day**				
Baseline (n = 5021)	71.6 (70.4, 72.8)	49.0 (48.1, 49.8)	21.9 (21.3, 22.6)	13.3 (12.9, 13.7)
Follow-up (n = 5021)	66.0 (64.7, 67.2)	46.8 (45.9, 47.7)	21.2 (20.5, 21.9)	13.2 (12.7, 13.8)
**Weekend, minutes/day**				
Baseline (n = 4556)	62.1 (60.4, 63.7)	43.0 (41.9, 44.2)	18.7 (17.9, 19.6)	12.1 (11.6, 12.6)
Follow-up (n = 4312)	52.2 (50.5, 54.0)	34.5 (33.1, 35.5)	17.2 (16.3, 18.2)	10.6 (9.9, 11.3)

MVPA, moderate- to vigorous-intensity physical activity; VPA, vigorous-intensity physical activity.

All analyses were weighted based on the sample size of each cohort.

Overall tracking of PA was examined using weighted Kappa statistics. Weighted Kappa coefficients showed fair agreement between baseline and follow-up in MVPA and VPA (Table 3). Compared to weekdays, weighted Kappa coefficients for weekends were lower.

**Table 3:** Weighted Kappa coefficients for baseline quintiles and follow-up quintiles of physical activity levels. Pooled analysis

	**MVPA**		**VPA**	
	**Boys**	**Girl**	**Boys**	**Girl**
	Weighted kappa (95% confidence interval)			
Weekday/weekend combined	0.28 (0.25, 0.30)	0.25 (0.22, 0.28)	0.27 (0.25, 0.30)	0.23 (0.21, 0.26)
Weekday	0.24 (0.21, 0.27)	0.24 (0.21, 0.26)	0.24 (0.21, 0.27)	0.22 (0.19, 0.24)
Weekend	0.22 (0.19, 0.25)	0.16 (0.13, 0.19)	0.18 (0.15, 0.21)	0.15 (0.12, 0.18)

MVPA, moderate- to vigorous-intensity physical activity; VPA, vigorous-intensity physical activity.

All analyses were weighted based on the sample size of each cohort.

The proportion staying in the same rank category of MVPA at follow-up was similar for active (≥ 80%tile of MVPA at baseline) and inactive (< 20%tile) children. Approximately 44% of boys and 40% of girls in low MVPA at baseline stayed inactive at follow-up. Approximately 41% of boys and 38% of girls in high MVPA at baseline fell into high MVPA at follow-up. The same proportion (17.5%) of the 20 to <80%tile group of MVPA at baseline changed to high MVPA or low MVPA at follow-up. However, the proportion staying in the same rank category of VPA at follow-up for children was somewhat gender-specific. Approximately 37% of each of boys and girls in low VPA at baseline had low VPA at follow-up, while approximately 45% of boys and 34% of girls in high VPA at baseline had high VPA at follow-up.

Table 4 presents adjusted ORs from the multinomial logistic regression models. Type of day-specific analysis indicated that weekday tracking was higher compared to weekend tracking. During the weekdays, active girls were less likely to stay in the same rank category when compared to inactive girls, active boys, or inactive boys (*Ps* < 0.05). When compared to boys within the 20 to < 80%tile of VPA at baseline, VPA tracking for boys with high baseline VPA was higher than boys with low baseline VPA over 1–3 years (ORs: 3.9 [95% CI: 3.1, 5.0] vs. 2.1 [95% CI: 1.6, 2.6]). In contrast to boys, among girls, high VPA was less stable when compared low VPA (ORs: 1.8 [95% CI: 1.4, 2.2] vs. 2.6 [95% CI: 2.1, 3.2]). The weekend tracking was statistically significant, but low, for both boys and girls, and particularly for girls with high VPA (OR: 1.3 [95% CI: 1.0 vs. 1.7]).

**Table 4:** Odds ratios to predict stability of physical activity levels. Pooled analysis

			**Weekday/weekend combined**		**Weekday**		**Weekend**	
	**Predictor**	**Outcome**	**Boys**	**Girls**	**Boys**	**Girls**	**Boys**	**Girls**
				Odds ratio (95% confidence interval)				
MVPA model	Age at follow-up (years)	<20%tile^a^	1.1 (1.1, 1.2)	1.1 (1.0, 1.2)	1.1 (1.0, 1.2)	1.0 (1.0, 1.1)	1.2 (1.1, 1.3)	1.1 (1.0, 1.2)
		20- < 80%tile^a^	Ref.	Ref.	Ref.	Ref.	Ref.	Ref.
		≥80%tile^a^	1.0 (0.9, 1.0)	0.9 (0.9, 1.0)	1.0 (1.0, 1.1)	0.9 (0.9, 1.0)	1.0 (0.9, 1.0)	0.9 (0.8, 0.9)
	Follow-up duration (years)	<20%tile^a^	1.1 (1.0, 1.3)	1.1 (0.9, 1.2)	1.1 (0.9, 1.3)	1.1 (0.9, 1.3)	1.3 (1.1, 1.6)	1.0 (0.9, 1.3)
		20- < 80%tile^a^	Ref.	Ref.	Ref.	Ref.	Ref.	Ref.
		≥80%tile^a^	0.9 (0.8, 1.1)	0.9 (0.8, 1.0)	1.0 (0.8, 1.2)	0.9 (0.8, 1.1)	0.8 (0.7, 1.0)	0.8 (0.6, 0.9)
	<20%tile^b^	<20%tile^a^	3.4 (2.7, 4.3)	2.6 (2.1, 3.3)	2.7 (2.1, 3.4)	2.8 (2.3, 3.6)	1.8 (1.4, 2.3)	1.9 (1.4, 2.4)
	20- < 80%tile^b^	20- < 80%tile^a^	Ref.	Ref.	Ref.	Ref.	Ref.	Ref.
	≥80%tile^b^	≥80%tile^a^	2.9 (2.3, 3.6)	2.4 (1.9, 3.0)	2.5 (2.0, 3.2)	1.6 (1.2, 2.0)	1.9 (1.4, 2.5)	1.7 (1.3, 2.2)
VPA model	Age at follow-up (years)	<20%tile^c^	1.0 (1.0, 1.1)	1.1 (1.0, 1.1)	1.0 (1.0, 1.1)	1.1 (1.0, 1.1)	1.1 (1.1, 1.2)	1.1 (1.1, 1.2)
		20- < 80%tile^c^	Ref.	Ref.	Ref.	Ref.	Ref.	Ref.
		≥80%tile^c^	1.0 (1.0, 1.1)	1.0 (0.9, 1.0)	1.0 (1.0, 1.1)	1.0 (0.9, 1.0)	1.0 (0.9, 1.0)	0.9 (0.9, 1.0)
	Follow-up duration (years)	<20%tile^c^	1.1 (0.9, 1.3)	1.0 (0.9, 1.2)	1.0 (0.9, 1.2)	1.1 (0.9, 1.3)	1.4 (1.1, 1.7)	1.2 (1.0, 1.4)
		20- < 80%tile^c^	Ref.	Ref.	Ref.	Ref.	Ref.	Ref.
		≥80%tile^c^	1.0 (0.8, 1.2)	1.0 (0.8, 1.1)	1.0 (0.8, 1.2)	1.0 (0.8, 1.1)	0.9 (0.7, 1.1)	0.9 (0.7, 1.0)
	<20%tile^d^	<20%tile^c^	2.4 (1.9, 3.0)	2.5 (2.0, 3.2)	2.1 (1.6, 2.6)	2.6 (2.1, 3.2)	1.8 (1.4, 2.3)	1.8 (1.4, 2.4)
	20- < 80%tile^d^	20- < 80%tile^c^	Ref.	Ref.	Ref.	Ref.	Ref.	Ref.
	≥80%tile^d^	≥80%tile^c^	3.9 (3.1, 4.9)	1.9 (1.5, 2.4)	3.9 (3.1, 5.0)	1.8 (1.4, 2.2)	1.8 (1.3, 2.3)	1.3 (1.0, 1.7)

^a^MVPA at follow-up; ^b^MVPA at baseline; ^c^VPA at follow-up; ^d^VPA at baseline.

MVPA, moderate- to vigorous-intensity physical activity; VPA, vigorous-intensity physical activity.

All analyses were weighted based on the sample size of each cohort.

Gender- and type of day-specific multinomial logistic regression model: follow-up [M]VPA (<20%tile, 20- < 80%tile, and ≥80%tile; reference group: 20- < 80%tile) = centered age at follow-up (years) + follow-up duration (years) + baseline [M]VPA (<20%tile, 20- < 80%tile, and ≥80%tile; reference group: 20- < 80%tile).

The confounding effects of age and follow-up duration were also presented in Table 4. In general, age was positively associated with being low PA. A longer follow-up duration was significantly associated with being low VPA at follow-up on weekend.

## Discussion

Using a large pooled sample of children and objectively measured PA data, this study compared the extent of PA tracking between active and inactive groups of children on weekdays and weekends during the behaviorally-important transition period of late childhood to early adolescence. Our large sample size allowed us to stratify the data by gender, type of day, and level of PA while maintaining sufficient study power. However, there are limitations to our work. For example, we were not able to account for the potential confounding factors such as race/ethnicity, socio-economic status, and seasonal variation [8]. With respect to seasonal variation, however, investigators for the studies used in our analysis attempted to collect baseline and follow-up PA data during similar seasons. In addition, the use of a secondary dataset did not allow us to examine original participation rates. A large loss to follow-up might introduce selection bias if the extent of PA tracking is different for those who completed PA measurements compared with those who did not. However, additional analysis excluding the CLAN and HEAPS datasets where a majority of participants with valid accelerometry data at baseline did not have valid data at follow-up revealed consistent findings with the current report. These limitations suggest caution when generalizing our study results.

Studies have reported low PA levels of children during weekend days vs. weekdays [8,19]. However, to the best of our knowledge, tracking data contrasting weekend PA to weekend PA have not been previously reported. We found that MVPA and VPA are moderately stable during weekdays, but not stable during weekends. Our results suggest that public health interventions which successfully build PA into the routine of school days may be able to sustain this behavior pattern as children move into adolescence. Future studies examining the determinants of children’s weekday vs. weekend PA are needed.

In our study, the stability of weekday VPA was greater among boys with high VPA vs. boys with low VPA. A comprehensive review of tracking by Malina [20] reported that sport participation tracks more strongly than other PA behavior, which would indirectly support our finding that high VPA in boys tracks better than low VPA. Assuming that sports provide much of the opportunity for weekday VPA, our results suggest that boys are sorted out of organized sport activities early in childhood possibly due to poor sport-related physical fitness or the inability to cross adolescent social circles.

Our findings on the PA tracking extent for girls are in an unhealthy direction. During weekdays, girls with low levels of PA remained inactive, while girls with high PA were more likely to change their PA behavior and became less active (relative to peers). Tracking of low PA and “untracking” of high PA are worrisome, given the known precipitous decline in PA by the great majority of girls during puberty. Our findings are somewhat inconsistent with Baggett et al. study [21], which reported similar levels of ORs for MVPA tracking (3.3 [95% CI: 2.3, 4.7]) and for inactivity tracking (3.6 [95% CI: 2.6, 5.2]), although it may be inappropriate to directly compare the results due to the different reference groups used. Similar to our current report, however, the IBDS investigators have recently shown a distinct (and troubling) tracking pattern for girls vs. boys [12]. Using survey data to examine tracking of sedentary behaviors from ages 5 to 13 years, the IBDS team reported that the ORs of playing ≥1 hour/day of video games at ages 8, 11, 13 if children played this amount at age 5 were 1.8 for boys and 3.5 for girls. These data indicate that the tracking of unhealthy PA behavior in girls begins early in childhood and continues into early adolescence [12]. Our finding of tracking of low PA and untracking of high PA in girls suggests a need to identify and target inactive girls early in childhood and to support active girls whose healthy behavior may change. In addition to testing interventions to increase PA in inactive girls, future research should examine the environmental support received by active girls to remain active. Recently, several studies have reported that girls prefer non-competitive PA [22,23]. We suspect that a traditional reliance on sports, which become more organized and competitive during adolescence, may be contributing to the low tracking of PA in active girls.

## Conclusion

The pooled analysis of five international cohort studies showed that the degree of PA tracking differs by gender and baseline PA levels: high tracking of inactivity among inactive girls and low tracking of PA among active girls. PA tracking was higher on the weekday than the weekend. The PA “routine” of weekdays should be used to help children establish healthy PA patterns. Interventions for untracking inactive children are recommended. Supports for PA increase and maintenance of girls are needed. In addition, behavioral reinforcement and a closer examination of the social environments that support PA may be needed for active girls to stay active over time.

## Abbreviations

ALSPAC, Avon Longitudinal Study of Parents and Children; CI, Confidence interval; CLAN, Children Living in Active Neighourhoods; EYHS, European Youth Heart Study; HEAPS, Healthy Eating and Play Study; IBDS, Iowa Bone Development Study; ICAD, International Children’s Accelerometry Database; MVPA, Moderate- to vigorous-intensity physical activity; OR, Odds ratio; PA, Physical activity; PEACH, Personal and Environmental Associations with Children’s Health; VPA, Vigorous-intensity physical activity.

## Competing interests

The authors declare that they have no competing interests.

## Authors’ contributions

SK has made substantial contributions to conception and design, analysis, interpretation of data, and manuscript drafting. KJ has been involved in drafting the manuscript and revising it critically for important intellectual content. A. Ness, MD, Avon Longitudinal Study of Parents and Children (ALSPAC), School of Oral and Dental Sciences, University of Bristol K. F. Janz, PhD, Iowa Bone Development Study, Department of Health and Sports Studies, Department of Epidemiology, University of Iowa, Iowa City A. Cooper, PhD, Personal and Environmental Associations with Children's Health (PEACH), Centre for Exercise, Nutrition and Health Sciences, University of Bristol, Bristol, UK J. Salmon, PhD; Children Living in Active Neigbourhoods (CLAN) and Healthy Eating and Play Study (HEAPS), School of Exercise and Nutrition Sciences, Deakin University, Melbourne, Australia. L.B.Sherar, PhD, School of Sports, Exercise and Health Sciences, Loughborough University, UK U Ekelund, PhD; Department of Sport Medicine, Norwegian School of Sort Science, Oslo, Norway and; 2) MRC Epidemiology Unit, Cambridge, UK.Dale Esliger, PhD, School of Sports, Exercise and Health Sciences, Loughborough University, UK Ashley Cooper, PhD, Centre for Exercise, Nutrition and Health Sciences, School for Policy Studies, University of Bristol, UK Pippa Griew, MSc, School of Sport and Health Sciences, University of Exeter, Exeter, UK,"
